# Perception of Ticks and Tick-Borne Diseases Worldwide

**DOI:** 10.3390/pathogens12101258

**Published:** 2023-10-19

**Authors:** José de la Fuente, Agustín Estrada-Peña, Marta Rafael, Consuelo Almazán, Sergio Bermúdez, Abdelbaset E. Abdelbaset, Paul D. Kasaija, Fredrick Kabi, Foluke Adedayo Akande, Dorcas Oluwakemi Ajagbe, Timothy Bamgbose, Srikant Ghosh, Azhahianambi Palavesam, Penny H. Hamid, Charlotte L. Oskam, Siobhon L. Egan, Amanda Duarte-Barbosa, Olcay Hekimoğlu, Matias P. J. Szabó, Marcelo B. Labruna, Ananta Dahal

**Affiliations:** 1 SaBio, Instituto de Investigación en Recursos Cinegéticos, IREC-CSIC-UCLM-JCCM, Ronda de Toledo 12, 13005 Ciudad Real, Spain; marta.simoes@alu.uclm.es; 2 Department of Veterinary Pathobiology, Center for Veterinary Health Sciences, Oklahoma State University, Stillwater, OK 74078, USA; 3 Department of Animal Health, Faculty of Veterinary Medicine, University of Zaragoza, 50013 Zaragoza, Spain; 4 Research Group in Emerging Zoonoses, Instituto Agroalimentario de Aragón-IA2, Universidad de Zaragoza-CITA, 50013 Zaragoza, Spain; 5 Facultad de Ciencias Naturales, Universidad Autonóma de Querétaro, Avenida de las Ciencias S/N Juriquilla, Querétaro 76230, Mexico; c_almazan_g@hotmail.com; 6 Medical Entomology Research Department, Gorgas Memorial Institute for Health Research, Panama City 0816-02593, Panama; sbermudez@gorgas.gob.pa; 7 Laboratory of Parasitology, Graduate School of Infectious Diseases, Faculty of Veterinary Medicine, Hokkaido University, Kita-18, Nishi-9, Sapporo 060-0818, Hokkaido, Japan; abdelbaset2006@hotmail.com; 8 National Livestock Resources Research Institute (NaLIRRI/NARO), Kampala P.O. Box 5704, Uganda; kpauldavis@gmail.com (P.D.K.); freddykabi@gmail.com (F.K.); 9 Department of Veterinary Parasitology and Entomology, College of Veterinary Medicine, Federal University of Agriculture, Abeokuta 111101, Ogun State, Nigeria; akandefa@funaab.edu.ng; 10 Department of Pure and Applied Zoology, College of Biological Sciences, Federal University of Agriculture, Abeokuta 111101, Ogun State, Nigeria; ajagbedorcas@gmail.com; 11 Department of Biological Sciences, Microbiology Unit, Faculty of Science, Kings University, Ode-Omu City 221102, Osun State, Nigeria; bamgbosetimothy@gmail.com; 12 Entomology Laboratory, Indian Veterinary Research Institute, Izatnagar, Bareilly 243122, Uttar Pradesh, India; sghoshtick@gmail.com; 13 IVRI-Eastern Regional Station, 37, Belgachia Road, Kolkata 700037, West Bengal, India; 14 Translational Research Platform for Veterinary Biologicals, Centre for Animal Health Studies, Tamil Nadu Veterinary and Animal Sciences University, Chennai 600051, Tamil Nadu, India; nambibio@gmail.com; 15 Department of Animal Science, Universitas Sebelas Maret, Surakarta 57126, Indonesia; pennyhumaidahhamid@staff.uns.ac.id; 16 School of Medical, Molecular and Forensic Sciences, Murdoch University, Perth, WA 6150, Australia; c.oskam@murdoch.edu.au (C.L.O.); siobhon.egan@murdoch.edu.au (S.L.E.); 17 Centre for One Health and Biosecurity, Harry Butler Institute, Murdoch University, Perth, WA 6150, Australia; a.duartebarbosa@murdoch.edu.au; 18 School of Veterinary Medicine, Murdoch University, Perth, WA 6150, Australia; 19 Division of Ecology, Faculty of Science, Hacettepe University, Beytepe, Ankara 06800, Turkey; olcayh@hacettepe.edu.tr; 20 Laboratório de Ixodologia, Faculdade de Medicina Veterinária, Universidade Federal de Uberlândia, Av. Pará, 1720/Campus Umuarama-Bloco 2T, Uberlândia 38400-902, Brazil; szabo@famev.ufu.br; 21 Faculty of Veterinary Medicine and Animal Science, University of São Paulo, Sao Paulo 05508-220, Brazil; labruna@usp.br; 22 Department of Microbiology and Parasitology, Faculty of Animal Science, Veterinary Science and Fisheries, Agriculture and Forestry University, Chitwan 44200, Nepal; dr.ananta.dahal@gmail.com

**Keywords:** tick, tick-borne diseases, environment, surveillance, epidemics, vaccine

## Abstract

In this comprehensive review study, we addressed the challenge posed by ticks and tick-borne diseases (TBDs) with growing incidence affecting human and animal health worldwide. Data and perspectives were collected from different countries and regions worldwide, including America, Europe, Africa, Asia, and Oceania. The results updated the current situation with ticks and TBD and how it is perceived by society with information bias and gaps. The study reinforces the importance of multidisciplinary and international collaborations to advance in the surveillance, communication and proposed future directions to address these challenges.

## 1. Introduction

Ticks and tick-borne diseases (TBDs) are a growing burden worldwide with (re)emerging diseases affecting human and animal health (e.g., recent references [1,2,3,4,5,6,7]). Factors behind this increase in cases, detection of new pathogens, or new epidemics in areas previously free of a pathogen are varied and sometimes of a local nature (i.e., [8,9]). For example, the trends of climate have been mentioned as the source of spread of some species of ticks [10,11]; the availability of meta-genomics improved the detection of previously unknown tick-borne viruses [12,13]. The changes in the landscape derived from human actions (e.g., changes in culture patterns, abandonment of culture areas, deforestation in some zones of South America) have been indicated as the main reason for epidemics of tick-borne pathogens in both animals and humans [14].

Hominids evolved in interaction with ticks and TBD as supported by fossil tick amber inclusions dated at ca. 100 Mya (Cretaceous), estimated origin of Ixodida at ca. 350 Mya, and the presence of TBPs in fossil ticks [15,16,17,18,19]. However, while some non-human primates have specific species of ticks, the same does not hold for species of ticks parasitizing the genus *Homo*, and *Homo sapiens* lacks its “own” species of ticks. All the species of ticks affecting humans (compiled by [20]) are either generalist species or the result of an accidental parasitism of ticks with varied specificity (ruminants, carnivores). On the other hand, the pathogens carried by these species affecting non-human primates have been seldom investigated, like the Kyasanur forest virus and *Haemaphysalis bispinosa*.

The conclusion is that both adequate passive and active surveillance, according to the logistic issues or other circumstances [21] regarding ticks affecting humans, and an active study of ticks affecting livestock and pets, aiming to improve both the health of the animals and the economic outcome, are necessary. However, despite advances in the surveillance, epidemiology, identification/diagnostics, and preventive/control interventions for ticks and TBD, major challenges are faced due to global expansion and increased incidence of TBD. One of these challenges is the difference that may exist in the perception of ticks and TBD worldwide. This perception is impossible to capture without the view of experts in different countries and regions.

**Figure 1:**
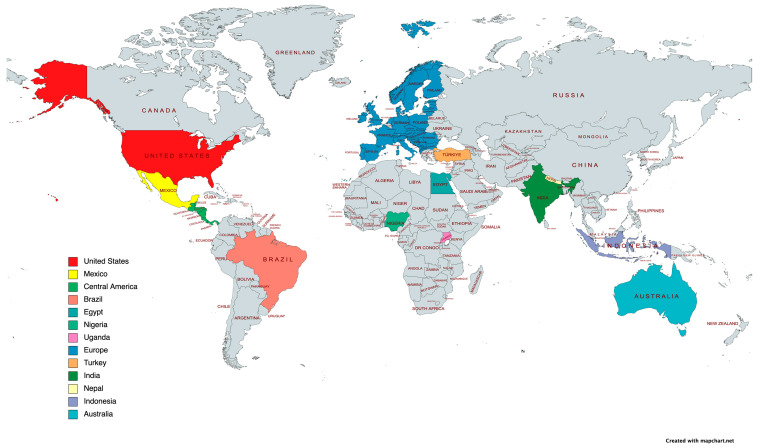
World map with the contributing countries and regions. Map created with mapchart.net (https://www.mapchart.net/world.html, accessed on 6 October 2023).

To address this challenge, in this comprehensive review study we provide analysis of information collected from contributions on ticks and TBD from different countries in multiple world regions (Figure 1). The results provided worldwide contributions on current situations with ticks and TBD, perception by different societal sectors, and the identification of information bias and gaps for future directions to address these limitations.

## 2. Contributions from Different Countries and Regions Worldwide

### 2.1. United States of America

Ticks and tick-borne disease constitute a growing burden in the United States (US) in both residential urban and land environments [10,22,23]. According to a recent publication by Eisen (2022) [10], 36 ixodid (most recorded, *Ixodes scapularis*, *Amblyomma americanum*, *Dermacentor variabilis*, *Ixodes pacificus*, and *Dermacentor andersoni*) and 13 argasid species (most recorded, *Otobius megnini* and *Ornithodoros coriaceus*) have been associated with human infestations in the US. Other tick species recorded in humans (>250 records) included *Ixodes cookei*, *Dermacentor occidentalis*, *Rhipicephalus sanguineus* s.l., *Dermacentor albipictus*, and *Amblyomma maculatum* [10]. The most recorded tick species in humans representing 67% of all ixodid tick records is *I. scapularis*, vector of pathogens and associated diseases, *Borrelia burgdorferi* senso stricto and *Borrelia mayonii* (Lyme disease), *Borrelia miyamotoi* (hard tick-borne relapsing fever), *Anaplasma phagocytophilum* (human granulocytic anaplasmosis), *Ehrlichia muris eauclairensis* (ehrlichiosis), *Babesia microti* (babesiosis), and Powassan virus (Powassan encephalitis) [10]. Even in Alaska, 15 tick species have been associated with human infestations, including historically found species (*Haemaphysalis leporispalustris*, *Ixodes angustus*, *Ixodes auritulus*, *Ixodes howelli*, *Ixodes signatus*, *Ixodes uriae*) and non-native species (*A. americanum*, *Dermacentor andersoni*, *D. occidentalis*, *Dermacentor variabilis*, *I. pacificus*, *Ixodes ricinus*, *I. scapularis*, *Ixodes texanus*, *R. sanguineus* sensu lato), some of which have not been associated with recent travels [24]. Main animal hosts include domestic animals, wild mammals, lizards, tortoises, and wild birds [10,24].

Factors such as climate change drive the expanding geographical range in the US of tick species such as *A. americanum* and *I. scapularis* and thus the incidence of TBDs such as anaplasmosis, babesiosis, Lyme disease, ehrlichiosis, and arboviral diseases [9,10,21]. The increased incidence of alpha-gal syndrome (AGS) has also been associated with expanding *A. americanum* [10] and is underdiagnosed [25,26]. AGS is an emerging multisymptomatic allergic disease mediated by IgE-type antibody response to galactose-alpha-1,3-galactose (alpha-gal) and associated with tick bites and consumption of mammalian meat and derived products containing alpha-gal [25,26,27].

Personal protection measures to prevent human contact with ticks and thus reduce the risk of tick bites are highly recommended to be used consistently [28,29]. According to Eisen (2022) [29], protection measures include “use of repellents, wearing untreated or permethrin-treated protective clothing, and conducting tick checks after coming inside, aided by removing outdoor clothing articles and running them in a dryer on high heat (to kill undetected ticks) and taking a shower/bath (to aid in detecting ticks on the skin)”. Other protection measures to consider include landscaping, vegetation management, tick host fencing, use of four-poster tick control deer feeders to apply acaracide to white-tailed deer, deer herd reduction, implementation of i-tree canopy vegetation cover subtype classification to predict peri-domestic tick presence, pet tick control, and interventions to kill host-seeking ticks or ticks infesting rodents [11,22,30,31,32,33,34]. However, although some of these measures are widely used, factors such as income, age, gender, race, and county of residence may affect the application of protection measures such as pesticides, and the correlation between protection measures and protective impact needs to be investigated for better public guidance [22,29,30,35].

Informing the population on the risks associated with ticks and TBD and targeted education for the implementation of protection measures through social media and advertisements is important to reduce the incidence of TBD [28,35]. Although the U.S. Department of Agriculture (USDA, Washington, DC, USA) and the National Institutes of Health (NIH, Bethesda, MD, USA) provide online free access information about TBD (Supplementary Dataset S1), gaps in population knowledge and differences in the attitudes and motivation such as forgetfulness, safety concerns, and lack of awareness affect the implementation of protection measures [36,37,38]. Regarding AGS, information available for healthcare providers and the general population is limited, supporting the need for surveillance and to provide guidelines for disease diagnosis and management [25,26,27]. The Centers for Disease Control and Prevention (CDC) provide online information on national tick and TBD surveillance programs ([39]; Supplementary Dataset S2). Nevertheless, implementation of effective surveillance using flag/drag tick samples, citizen science, and smartphone applications such as The Tick App is important to collect updated information [36,40,41,42]. Additionally, the communication between people with disease symptoms after tick bites and healthcare providers is important to improve surveillance, diagnostic, and treatment measures [43].

Gaps in the diagnosis, prognosis, treatment, and prevention of TBDs are a limitation for the reduction of the incidence and severity associated with these diseases [44]. Laboratory diagnostic methods are not well implemented and not effective for diagnosis during the acute illness stage when timely treatment is needed, while nucleic acid amplification tests are most effective [45]. To address these limitations, the ehrlichiosis and anaplasmosis subcommittee report to the Tick-borne Disease Working Group “identified the needs to develop sensitive, specific acute stage diagnostic tests for local clinical laboratories and point-of-care testing, to develop approaches for utilizing electronic medical records, data mining, and artificial intelligence for assisting early diagnosis and treatment, and to develop adjunctive therapies for severe disease” [45].

### 2.2. Mexico

Ticks and tick-borne diseases are a significant concern in Mexico. The two most common tick species affecting domestic animals in the country are the hard ticks *Rhipicephalus microplus and R. sanguineus. R. microplus* is found in over 60% of Mexico, while *R. sanguineus* is more widely distributed [46,47]. Other tick species such as *Amblyomma* spp., *Dermacentor* spp., and *Ixodes* spp. can also be found. *Otobius megnini* has been frequently found parasitizing cattle and less regularly found on dogs and horses [48,49].

Babesiosis and anaplasmosis are the most prevalent TBDs in cattle, with prevalence rates ranging from 2% to 94% and >50%, respectively [50,51]. These diseases constantly threaten livestock and limit beef cattle’s genetic improvement due to the high morbidity and mortality of high-value animals introduced to tick-infested areas [50]. Equine babesiosis and theileriosis are prevalent in horses, complicating their movement and transportation for sport, competition, and as companion equids [51]. In dogs, TBD information is primarily based on commercial diagnoses performed by veterinarians. Canine babesiosis has been documented since the last century. Still, molecular identification of *B. vogeli* was performed recently [52], as well as that of *Ehrlichia canis, Anaplasma platys*, and *A. phagocytophilum* [48,53].

Rickettsiosis by *R. rickettsii* is the most important TBD in humans in northwestern Mexico, with mortality rates of 30–40% [54]. In addition, infection with *B. burgdorferi* has been confirmed in over 100 cases [55]. Although evidence of human babesiosis and anaplasmosis has been documented for a long time, only recently have *B. microti* and *A. phagocytophilum* been molecularly identified [56,57]. However, the tick vector remains to be determined.

Cattle producers, especially those in northern Mexico and the Gulf of Mexico, know the importance of *R. microplus* due to the national campaign against this tick. However, a wide gap in education and training for other tick species still needs to be addressed. Therefore, misunderstanding and need for knowledge on the role of ticks as vectors of pathogens of zoonosis concern in both rural and suburban areas exist. Outbreaks of TBD during the season with the highest abundance of brown dog ticks in the northwest of the country are an example of the lack of information on preventing tick infestations and controlling TBD.

The prevention and control of ticks and the diseases they transmit to animals and humans require a research agenda that considers tick biology, integrated tick control, and science-based use of acaricides alone and combined with anti-tick vaccines together with management of wild animal translocation, tick surveillance, identification of tick carriers and reservoirs, standardization of diagnosis methods, and molecular identification of pathogens [58].

Regarding TBD affecting humans, training programs on tick identification, prevention, and control measures are required to avoid seasonal outbreaks of rickettsiosis and other diseases. Also, studies to demonstrate the transmission of some pathogens, such as *B. microti, B. burgdorferi,* and *A. phagocytophilum,* in association with reservoirs, tick carriers, and origin of infection need to be carried out [58]. All these actions require close collaboration between veterinarians, researchers, public and animal health authorities, wildlife specialists, and other stakeholders.

### 2.3. Central America

Central America contains an approximate area of 522,000 km^2^ integrating a wide biological diversity, which includes a rich fauna of ticks, with about 80 species reported to date (Supplementary Dataset S3). Of this diversity of ticks, *Amblyomma mixtum*, *Amblyomma ovale*, *Dermacentor nitens*, *R. microplus*, *Rhipicephalus sanguineus* s.l., *Alectorobius puertoricensis*, and *Alectorobius talaje* have been reported as relevant to human and animal health due to their role as vectors of pathogens causing rickettsiosis, ehrlichiosis, anaplasmosis, relapsing fever, and babesiosis, as well as causing paralysis and severe allergies.

Central America has a long-standing history in relation to cases of human and animal parasitism, with the first records of effects on humans being recorded in the 19th century in Guatemala (*Ornithodoros talaje* and *Amblyomma sabanerae*). At the beginning of the 20th century, the first reports of clinical cases of tick-borne pathogens in humans were reported in Panama (*Rickettsia rickettsii* relapsing fever and spotted fever) and Costa Rica (*R. rickettsii* spotted fever). In fact, rickettsiosis is the most important group of diseases reported in Central America, since there are confirmed fatal cases in these two countries, in addition to reports of severe rickettsiosis in acute patients from Guatemala, Honduras, and Nicaragua. To date, there are close to 15 *Rickettsia* species or strains reported in Central American ticks, which makes it the most studied and reported genus of bacteria in the region. Of these, there is no information about their relevance in public health in species such as *R. amblyommatis, R. bellii*, or the rickettsial endosymbiont of *Ixodes* spp., *Candidatus* “R. colombianensi”.

Other microorganisms detected in ticks from Central America include the genera Ehrlichia (*E. canis*, *E.* cf. *chaffeensis*), Anaplasma (*A. marginale*, *A. phagocitophylum*, *A. platys*), Borrelia (*B. puertoricensis*, Borrelia burgdorferi group), and hemoparasites like Babesia (*B. odocoilei*, *B. vogeli*) and Hepatozoon (*H. canis*, *Hepatozoon* spp). There is also serologic evidence of ehrlichiosis, anaplasmosis, and babesiosis in domestic animals; a probable case of canine ehrlichiosis in a boy from Panama and serology compatible with ehrlichiosis in human blood from Costa Rica. Finally, studies of the microbiome have been developed in Nicaragua and Panama, that revealed several genera of bacteria in ticks.

### 2.4. Brazil

Brazil is a vast and ecologically diverse country with several distinct biomes that include tropical forests (Amazon and Atlantic rainforest), savannah (Cerrado), grasslands (Pampa), semi-arid (Caatinga), and the world’s largest tropical wetland (Pantanal). These biomes are characterized by their unique climate, vegetation, and wildlife. However, huge areas within each biome were transformed into anthropogenic landscapes to become part of the Global Human Ecosystem. This ecological mosaic has shaped the current Brazilian tick fauna and associated microbiota, but general knowledge about most tick species is lacking, and epidemiological data about transmitted pathogens are also scarce. Indeed, knowledge of tick-borne diseases is primarily related to those agents with a major impact on human welfare.

The tick fauna of the country is by now composed of 78 species, 53 Ixodidae and 25 Argasidae. *Amblyomma* remains as the richest, with 34 valid species [59]. The original tick fauna was modified by the introduction of exotic species, outstandingly *R. microplus* and two species of the *Rhipicephalus sanguineus* complex [60,61,62]. Additionally, a profound alteration in the distribution of and probably density of various tick species indigenous to the neotropical region occurred and tick-borne pathogenic microorganisms probably followed the same trend. However, these changes are hard to evaluate because base-line values of the original situation are lacking for comparison.

Undoubtedly, the cattle tick *R. microplus* is a species that raises an important level of apprehension. It is the species most associated with economic losses throughout a country that had a commercial herd estimated at 224.6 million heads in 2022 [63]. In the last broad assessment of the negative impact of the cattle tick, an annual loss of USD 3.24 billion was estimated [64]. This loss includes the negative impact of infections caused by the major cattle tick-borne pathogens *Babesia* spp. and *Anaplasma marginale* and the disease commonly known as “Bovine parasitic sadness” [65]. One major concern is the occurrence of *R. microplus* tick populations with multiple drug resistance since field control is performed almost exclusively by the application of chemical acaricides [66,67].

The main horse ticks in the country are *Amblyomma sculptum* and *Dermacentor nitens* (named previously *Amblyomma cajennense* and *Anocentor nitens*) [68]. Among tick-borne diseases, equine piroplasmosis caused by *Babesia caballi* and *Theileria equi* infection is enzootic in Brazil [69,70]. Several tick species are supposed to transmit these pathogens but *D. nitens* is considered the sole vector of *B. caballi*, while *T. equi* is transmitted by *R. microplus* and possibly by *A. sculptum* [70,71]. Curiously, sheep and goats in Brazil are not primary hosts for ticks and are usually parasitized when sharing pastures with other tick-infested animals such as bovines or horses [72].

In relation to dogs, the anthropogenic landscapes throughout Brazil are widely colonized by ticks of the *R. sanguineus* complex with a wide distribution in anthropized areas of the country [61]. These species, particularly the tropical lineage (recently considered *Rhipicephalus linnaei* [73]), are vectors of *Ehrliquia canis* and *Babesia vogeli,* the agents, respectively, of canine monocytic ehrlichiosis and canine piroplasmosis, collectively known by pet owners as the “tick disease” (in Brazilian Portuguese, “doença do carrapato”). Both *E. canis* and *B. vogeli* have been widely reported in dogs from Brazil [74,75]. These tick species have been found infected with *R. rickettsii* [76], nonetheless human rickettsiosis caused by infected tick bite remains elusive. On the other hand, *Amblyomma aureolatum,* a species restricted to the Atlantic rainforest and the Pampa biome in the south of the country [77,78], is the natural vector of *Rangelia vitalii*, the etiologic agent of canine rangeliosis, the most severe canine piroplasmosis of the western hemisphere [79]. Although *R. vitalii* is highly pathogenic to domestic dogs, it is not pathogenic or is much less pathogenic to one of its natural hosts, the crab-eating fox *Cerdocyon thous* [80]. Dogs in Brazil may also be infected by Hepatozoidae species (e.g., *Hepatozoon canis*), usually causing a mild disease [81]. The epidemiology of the infection in wild and domestic animals caused by Hepatozoidae species is not yet fully understood in Brazil, moreover, new species are being reported [82,83].

Dogs are also involved in the epidemiology of human tick-borne rickettsiosis and should be considered a target species for tick control. Only two tick-borne *Rickettsia* species have been proven to cause human disease in Brazil, *R. rickettsii*, causing a frequently lethal disease, and *Rickettsia parkeri* strain Atlantic rainforest, responsible for milder non-lethal rickettsiosis [84]. Circumstantial evidence indicates that there is in the country a third and mild rickettsiosis caused by *Rickettsia parkeri* stricto sensu [85]. Wild carnivores are hosts for the adult ticks of *Amblyomma aureolatum*, *Amblyomma ovale*, and *Amblyomma tigrinum* and domestic dogs are common alternative hosts [86]. These tick species have been shown to be infected with, respectively, *R. rickettsii*, *Rickettsia parkeri* Atlantic rainforest strain, and *Rickettsia parkeri* sensu stricto and emerging knowledge indicates that dogs may bridge the infected ticks to human households [85,87,88]. Whereas *Rickettsia*-infected *A. ovale* ticks have a wide distribution within the country, infected *A. tigrinum* were detected only in the southern region [85]. Reports of human rickettsiosis due to *A. aureolatum* bites are more frequent in the southern part of the São Paulo metropolitan area, which has margins intermingled with forest remnants of the Atlantic rainforest [89].

*Rickettsia rickettsii* infection is the major human tick-borne disease in Brazil, the “febre maculosa Brasileira” (Brazilian spotted fever). Although the disease has a low prevalence and is overwhelmingly restricted to specific areas, it has gained significant attention and apprehension in society due to its high lethality. In fact, timely and correct antibiotic treatment is curative. Unfortunately, early diagnosis is not easy and relies on epidemiological data (febrile individuals bitten by ticks in endemic areas) since laboratory diagnosis is typically confirmatory after the recovery or death of those who are ill [90]. While rickettsiosis caused by infected *A. aureolatum* bites has been reported in the São Paulo metropolitan area [89], the primary and most widespread epidemiology for human *R. rickettsii* rickettsiosis in Brazil are infected *Amblyomma sculptum* larva and nymph tick bites [91] that have previously had blood meals on bacteremic capybaras (amplifying hosts, see [92]). Even though *A. sculptum* tick populations primarily feeding on capybaras are common along river and lake banks in southeast and midwest Brazil, endemic areas are mostly limited to southeast Brazil, particularly in anthropized areas within the former Atlantic rainforest biome [91,93,94].

A controversial tick-borne disease in Brazil is Lyme borreliosis. Lyme-like disease has been diagnosed since 1992 [95,96]. The disease is routinely diagnosed based on clinical and serological data and records of suspected cases included in the Brazilian Ministry of Health database [96]. There are also occasional molecular identification reports of bacteria from the *Borrelia burgdorferi* sensu lato complex [97]. However, *Borrelia burgdorferi* has not yet been isolated either from humans or ticks [93,94] and the ecological background to sustain its epidemiology within the country is weak. Only ticks belonging to the hard tick genus *Ixodes* have been shown to be competent vectors for the agent of Lyme disease, and among these, those of the *I. ricinus* complex [98]. Currently, there are 12 *Ixodes* species in Brazil [99] and only one, *Ixodes fuscipes* (previously *Ixodes aragaoi*), belongs to the *I. ricinus* complex. None of them are recognized as human parasites. Further, based on criteria for Lyme disease diagnosis proposed by the CDC of the United States, serological evidence of Lyme borreliosis in Brazil could be considered non-existent [96]. Very likely, the great number of diagnoses is related to the importance of the disease in the USA and great influence of American medical literature on Brazilian physicians. In the last decade, DNA of other potentially pathogenic *Borrelia* species, notably of the relapsing fever group, has been found in several hard and soft tick species throughout the country [100,101,102]. Some of these *Borrelia* have also been isolated from human-biting soft ticks, *Ornithodoros* spp. [75]. The relevance of these *Borrelia* species for public and animal health remains undetermined and warrants attention and additional studies.

Viruses are also significant tick-borne agents, and tick-associated viruses have already been documented in Brazil [103,104]. However, their role as pathogens remains uncertain. Indeed, there are numerous molecular studies reporting other potential tick-borne pathogens in ticks collected from domestic animals, wild animals, and the environment in Brazil. Still, these pathogens have not been definitively linked to infections and diseases. It remains to be established whether these entities will become important pathogens or will remain as components of a harmless microbiota.

Finally, it is noteworthy that in Brazil, like in other Latin American tropical countries, mosquito-borne diseases are at the top of the media and academic agenda in relation to all knowledge about vector-borne diseases affecting humans [105]. In contrast, human tick-borne diseases, caused by a great variety of viruses, bacteria, and protozoa, are arguably the most prominent in the United States and Europe [106]. This scenario seems incoherent if one considers that Brazil’s tick fauna is as diverse as the tick fauna of the United States or Europe [107]. Given the historical discrepancy in investments in science and technology between the northern and southern hemispheres of the planet, it is to be expected that many tick-borne diseases will emerge in Brazil in the coming decades as studies progress.

### 2.5. Europe

Ticks are an important part of the parasitic burden affecting livestock in Europe, as well as a growing issue regarding human health because of the transmission of pathogens. In Europe, prominent species of ticks affecting domestic animals (with even 6–7 generations per year, like *R. microplus* in many parts of the world) are absent, but reported species also represent an important burden in animal husbandry. The panorama is a wide repertoire of species, most of them affecting livestock, that colonize areas with very different environmental conditions therefore resulting in a “mosaic” of distribution [108,109,110,111,112], with different seasonal activity periods, ability to transmit different types of pathogens, and capacity to spread throughout the wild fauna of a region.

Most of these ticks have generalist feeding habits, affecting notably domestic ruminants and horses under extensive management. They constitute a large burden affecting the production of meat or milk, debilitating the animals and/or increasing abortions, favoring poor health conditions, and promoting the development of secondary diseases caused by opportunistic bacteria [111]. An additional issue is the use of acaricides against ticks, which contribute to contamination by these toxic products and the increase in the costs of management of the animals. It is important to note that most (if not all) species of ticks affecting livestock are shared with wild ungulates. Therefore, due to the co-existence of wildlife and livestock in large European regions, efforts to control or eradicate ticks are challenging. As in other regions, ticks prevail in nature through cycles of infestation affecting either domestic or wild ungulates as adults, with the immatures feeding commonly on many species of birds and rodents [112]. These feeding preferences are responsible for the maintenance and transmission of several pathogens of clinical importance. There is not a specific pattern of parasitism by ticks on animals in Europe. Most species of ticks, like *I. ricinus*, *Haemaphysalis punctata*, *Rhipicephalus* spp., *Hyalomma marginatum*, or *Dermacentor* spp., are true generalists and therefore will readily feed on a wide range of ruminants or carnivores [113]. These ticks are vectors for the transmission of protozoans like *Babesia* and *Theileria* and bacteria like *Anaplasma* spp. (different species following a clear latitudinal gradient), *Borrelia* spp., *Ehrlichia*, or *Neoehrlichia* [8]. Probably the most important virus transmitted by ticks in Europe belongs to the complex of strains of Flaviviridae tick-borne encephalitis virus, with a growing importance of the bunyavirus Crimean-Congo hemorrhagic fever virus (CCHFV).

Some species of ticks affecting livestock in Europe are also parasites of humans, and the pathogens carried by them may also be infectious agents of humans. Therefore, ticks in Europe have a double interest: the management of livestock to reduce their impact and their importance in producing disease in humans in the target area.

Europe can be roughly divided, according to latitude, into three regions, Mediterranean, Central, and Northern regions. The Mediterranean region is populated by species of ticks with an obvious seasonality because of the seasonal nature of the weather in the region. The most important species belong to the genera *Rhipicephalus* and *Hyalomma*. Due to the vegetal characteristics of the region, sheep and goats are the main livestock present in the area (high humidity deficit, high temperature), making these species the main vectors for several species of protozoans, like *Babesia* spp. and *Theileria* spp., or bacteria like *Anaplasma* ovis or *Rickettsia* spp. Many wild and domestic animals have high rates of positive serology against Rickettsia, but these bacteria have clinical significance in humans. However, the economic losses associated with acute infections of *Babesia* or *Theileria* constitute a serious burden for livestock management due to not only the treatment costs but also the lack of coordinated strategies to control the ticks or the insidious chronic infections that may devastate the economy of local (and small) farmers. In the absence of a coherent pattern of tick control, extensive farming rests on the criteria of the farmers, which are commonly far from scientific criteria.

Central Europe, including the British Isles and southern parts of Scandinavia and Finland, is the major area of distribution of the prominent species *I. ricinus* and *H. punctata*. These two species tend to concentrate on ruminants and are the main vectors of several species of protozoans of the genus *Babesia* and the bacterium *Anaplasma phagocytophilum*. Both pathogens are responsible for a wide array of clinical presentations, from the chronic one, with a course of abortions and serious weight loss, to the acute cases, in which death may be fast, even in 72 h. No efforts to determine the economic losses produced by these protozoans or bacteria have been addressed. Furthermore, the trends of climate in the region are pushing some species of both *Rhipicephalus* and *Hyalomma* to slowly spread into these central parts of the European continent [113]. This promotes (a) new species and new pathogens affecting livestock, (b) new seasonal patterns, previously unknown to veterinarians, that greatly complicate the control using synthetic acaricides, (c) new species of pathogens that could potentially affect humans (i.e., *Rickettsia* spp.). Northern Europe is usually too cold to host permanent populations of ticks. Only *I. ricinus* extends along the coasts of Norway [114], large parts of central Sweden [115], and some portions of southern Finland [116]. North of these areas, no ticks affecting livestock and/or humans have been reported; however, an area of colonization by populations of *Ixodes persulcatus* exists in Finland.

Most, if not all, species affecting livestock and pets in Europe may bite humans with a different pressure according to the climate gradient associated with the territory and with pathogen transmission. The pathogens carried by ticks are supported by populations of wild vertebrates, that have a different prominence according to the composition of the community of vertebrates [117]. According to the area of the territory, these associations of ticks–hosts (reservoirs) may change and therefore the array of transmitted pathogens may be different. Europe has a deep awareness of ticks and tick-borne pathogens affecting humans and several projects, programs, websites, and applications developed for cellphones exist to inform, prevent, and map the distribution of the species through so-called “citizen science”. Even with the many gaps that this kind of passive surveillance may have [118], the information is gaining a prevailing role in the European panorama. The European Centre for Disease Prevention and Control curates updated information on active surveys carried out by specialists. This results in a high awareness in most European countries, which, interestingly, is lower in Mediterranean countries because of the extra burden of mosquitoes and sandflies. This general degree of awareness and coordinated actions against ticks affecting humans is only comparable to that existing in the United States or in localized parts of Canada (those invaded by *Ixodes scapularis*) and seems to be absent in the rest of the world.

Other than pets, for which harmonized guidelines about tick control exist and owners commonly follow recommendations by veterinarians, there are no agreed protocols for tick control in Europe. Control (or attempt at eradication) of ticks depends on the perception of the owners, the recommendation of field veterinarians, and the availability of adequate acaricides, which may have different regulations depending on the country. The lack of harmonized protocols prevents the necessary joint effort to eradicate ticks. Also, the fact that the cycle of many tick species rests in their ability to parasitize wild animals (either large ungulates, rodents, or birds) further complicates the control of tick populations, because the treatment currently focuses only on livestock. Wild animals are commonly ignored, and even if addressed, the logistic difficulties for the control of ticks are formidable.

It is necessary to evaluate innovative control methods of ticks affecting livestock. This would not only reduce the burden of ticks and the derived economic costs but also help to prevent the transmission of pathogens to humans. However, this should be based on elaborated plans, agreed by many countries, tailored for the most important species affecting livestock, and adapted to the different peculiarities according to the target region.

### 2.6. Egypt

In Egypt, animal trade, climate, and anthropogenic factors contribute to the spread of tick species and TBD. The spillover of various tick-borne pathogens is likely to occur from sub-Saharan Africa and other Mediterranean basin countries. The development of acaricide resistance further exacerbates the widespread presence of ticks and TBD, posing a significant economic challenge in Egypt [119,120]. To date, eight tick species from the family Argasidae and forty-four species from the family Ixodidae have been reported, with *Hyalomma* sp. and *Rhipicephalus* sp. being the most common. Among TBD, anaplasmosis, babesiosis, theileriosis, and Q fever are frequently observed in livestock. In contrast, tick-borne zoonoses are underreported and likely underestimated, with a few studies in humans documenting anaplasmosis, borreliosis, Q fever, tick-borne rickettsiosis, babesiosis, Alkhurma hemorrhagic fever, and CCHF [119,120].

Enhancing the understanding of ticks and their associated diseases among various societal sectors, as well as the general population, is crucial for implementing effective control measures. In Egypt, ticks and TBD have predominantly been viewed through the lens of agricultural production rather than human health. Farmers’ perception is limited due to the widespread use of acaricides to eradicate ticks infesting animals, leading to the development of acaricide resistance [121,122]. Veterinarians’ awareness of major TBDs affecting livestock is high. However, awareness regarding human-biting ticks, associated zoonotic pathogens, and the use of tick repellents is lacking, particularly in rural areas. Healthcare facilities have insufficient diagnostic capacity to screen and report TBD. The absence of surveillance data on zoonotic TBD hampers the understanding of their distribution and burden among populations and public health professionals [119,120].

Considering the close interaction between humans, animals, and tick vectors, a multidisciplinary approach linking human, animal, and environmental health within a “One Health” framework is essential. Systematic and comprehensive surveillance studies investigating ticks and TBD in defined areas are needed. Collaborative efforts between Egypt and Europe, combining fieldwork, research capacity, and funding, could lead to a better understanding of the epidemiological landscape of ticks and TBD. This collaboration could also help establish a robust database. Furthermore, it is crucial to strictly monitor and control the influx of potentially infected animals and exotic tick species through animal trade [119,120].

### 2.7. Uganda

In Uganda, ticks including *Rhipicephalus appendiculatus, Rhipicephalus decoloratus*, *Amblyomma variegatum*, and *Rhipicephalus evertsi evertsi* are the most economically important ticks that parasitize cattle and transmit deadly disease pathogens. The key tick-borne disease pathogens are *Theileria parva, Babesia bovis*, *B. bigemina*, *A. marginale*, and *Ehrlichia ruminantium* whose infections result in high morbidity and mortality if naïve cattle become infected. These diseases and the tick vectors cause annual losses of USD 1.1 billion, thus affecting cattle-keeping communities in poverty.

However, the current tick control approaches mainly depend on the use of acaricides applied at a frequency of one to three times a week depending on the extent of the tick burden. The increasing frequency of application is critically indicative of acaricide-resistant tick genotypes [123].

The specific deleterious effects of tick infestations are bites which damage the hides in animals with high tick loads, blood loss and thus anemia, allergy due to toxins in tick saliva, chronic stress, and continuous irritation affecting animal welfare, leading to immuno-depression and loss of energy [124]. Specific economic losses result from failure to rear high-grade cattle due to their being highly susceptible to ticks and tick-borne diseases, death, abortion, poor-quality hides, treatment losses, and stunted growth, leading to delayed attainment of market weight.

Generally, more than 70% of Uganda’s population depends on agriculture for their livelihood, and the animal industry accounts for 17% of the national gross domestic product. Cattle farmers perceive ticks and tick-borne diseases as a big limitation to commercial livestock farming given the fact that transmitted pathogens limit the breeding of high-yielding cattle. The overdependence on acaricide for tick control is no longer viable, thus demanding the development of novel control strategies.

For public health purposes, ticks in Uganda are known to vector several pathogens of public health concern such as CCHF transmitted mainly by ticks of the genus *Hyalomma*. The virus could be circulating within Uganda silently, another reason for developing a novel tick control product, since it is known that livestock can support large populations of *Hyalomma* spp.

The proposed future directions for controlling ticks and tick-borne diseases will mainly rely on the integration of modern vaccine technology with the capacity to stimulate high immunity, continuous farmer education, and modern livestock management practices.

### 2.8. Nigeria

In Nigeria, the study of ticks and tick-borne diseases has a history spanning several decades [125,126,127,128]. Although there was a period without tick research, there has been a recent resurgence of interest in investigating various tick-borne pathogens in Nigeria [129,130,131,132,133,134,135] and in collaboration with five other African countries [136,137]. Various studies have reported on the distribution of ticks on different animal hosts across the country, including wild game animals and cattle that enter through trans-border routes [130,131,132,138,139,140,141,142,143,144]. Southern and northern Nigeria have recorded the presence of tick genera such as *Boophilus*, *Amblyomma*, *Rhipicephalus*, *Haemophysalis*, *Aponomma*, and *Hyalomma* [130,145,146,147]. The factors contributing to tick distribution in Nigeria include unrestricted animal cross-border movement, nomadic or trans-animal movement, the lack of strong quarantine regulations, widespread livestock grazing, and favorable climatic conditions.

Regarding tick-borne diseases, babesiosis, anaplasmosis, theileriosis, and ehrlichiosis have received the most attention in documented cases [145,146]. However, conditions like CCHF and African tick-bite fever have been underreported, possibly indicating a gap in surveillance and reporting mechanisms for these particular diseases. Also, hundreds of ticks have been gathered from a snake kept in a zoo. All the ticks harvested were *Amblyomma latum* of both sexes and at different stages of development. *Hepatozoon phythonis* was identified by thin blood smear from the same snake, while *Amblyomma tholloni* was found on an elephant calf that was to be kept in a private zoo in the state of Edo, Nigeria.

Many peri-domestic veterinary diseases have zoonotic potential. However, there is limited information on the prevalence and clinical outcome of tick-borne diseases in humans, despite their significant impact on pets, service dogs, and livestock. Additionally, there is no proper understanding of the diversity and expanse of pathogens that can be vectored by ticks. In some instances, people bitten by ticks in a university community have been prescribed only with pain relievers at the campus clinic, highlighting the inadequate attention given to tick bites and potential associated diseases. Among hunters, ticks are well recognized, but there is a lack of awareness regarding their role as vectors of pathogens and the diseases they might transmit (unpublished data). Similarly, foresters are aware of ticks but do not fully grasp the importance of using protective clothing or seeking testing after tick bites.

Previous studies have been restricted to specific geographic locations, thus there is a need for a nationwide extensive survey of different animals, including wildlife, offered for sale in some areas, for the diversity of ticks and tick-borne pathogens in animal and human populations. Awareness about the impact of ticks and tick-borne pathogens on various group of people, especially the at-risk groups like farmers, hunters, foresters, and veterinarians who serve as “middlemen” between the wild and domestic interphase, should be prioritized. The issue of acaricide resistance [148] which impacts both livestock and humans must be adequately addressed [149]. There are reports of pastoralist communities that directly spray their animals with herbicides and other pesticides not designed for animal use, therefore ignoring the possible toxic bioamplification, as well as the residues in milk and meat to be consumed, or the contaminating effects in water bodies and the environment.

### 2.9. India

In India, 109 tick species are reported to infest animals [150]. The tick index or tick burden in cattle was reported to be 0.922 to 1.0 [151,152] and the species diversity was high in rural parts in comparison to urban areas. As per a recent estimate, the TBD in animals is causing an economic loss of USD 787.63 million/year. Besides animals, several tick species such as *Amblyomma integrum*, *Haemaphysalis spinigera*, *Dermacentor auratus*, *Hyalomma isaaci*, *Rhipicephalus haemaphysaloides*, *R. sanguineus* s.l., and *Otobius megnini* are reportedly infesting human beings [153,154]. The use of acaricides by swabbing, dipping, spraying, pour-on, spot-on, and injection is the sole approach adopted for tick control. Awareness on environmental tick control or off-the-host tick control is lacking. No commercial anti-tick vaccines against the major cattle tick *R. microplus*/*H. anatolicum* are available which could be attributed to the diversity of targeted antigen sequences across the tick isolates in India [155]. Two phyto-acaricide technologies have been developed and approved by regulatory authorities and commercialized [156]. Multiacaricide-resistant *R. microplus* ticks pose a great threat to the dairy industry and warrant a newer class of acaricides in the near future [157]. A positive correlation between the tick burden on household cattle and resistance factor (R = 0.66) indicated a high level of acaricide resistance in animal production systems [152].

The TBDs affecting humans are Kyasanur forest disease (KFD), CCHF, Ganjam virus (GANV), Bhanja virus (BHAV), Lyme disease, Q fever, rickettsial infections (*Rickettsia conorii* and *R. rickettsii*), and babesiosis (*Babesia microti*) [158]. Meanwhile, animals suffer from theileriosis, babesiosis, anaplasmosis, ehrlichiosis, hepatozoonosis, and lumpy skin disease virus. There are only two licensed vaccines available against TBD in India, viz., tropical bovine theileriosis (Rakhsavac-T) and KFD for humans. Chemotherapy is the only option being practiced, controlling major TBD infections. However, studies on drug resistance in tick-borne pathogens are non-existent. So far, no nationwide systematic study has been undertaken to estimate the prevalence of TBD in humans and animals.

In rural communities, tick infestation is considered as one of the many problems animals have to suffer perennially and that is managed by washing animals, by rubbing them with dry fodder, or by the application of available acaricides at the local market when infestation is visible. In the organized sector, highly tick-susceptible cross-bred animals are maintained for higher production. The problem of ticks is regularly treated by the use of acaricides but without a strict adherence to the recommendation of the manufacturers. Although farmers are well aware of the high cost involved in the treatment of TBD, due to limited knowledge of the methods for tick control, resource-poor farmers face severe economic distress. On the other hand, resource-rich farmers are overusing anti-tick chemicals and thus resistant ticks are widespread. Since TBDs are reported only from some regions of the country, most animal owners do not give importance to the diseases caused by tick-borne pathogens. Pet owners lack knowledge on ectoparasites and are unable to differentiate ticks, lice, and fleas, and are completely unaware of the vector potential of zoonotic pathogens transmitted by ticks.

The future directions to control TBD should be focused on (a) a national acaricide resistance-monitoring system, (b) effective multicomponent and cross-protective anti- vaccines against important tick vector species and TBD using advanced vaccine platform technologies, (c) cryodesiccation technology to be standardized for the storage of live *Theileria annulata* vaccine, (d) establishment of a stock of TBD-resistant animals using genome-editing technologies, (e) promotion of natural effective anti-tick products for resistant tick management, and (f) training, capacity building, up-skilling, and awareness creation on the control of ticks and TBD.

### 2.10. Nepal

In the Nepalese context, there are inadequate studies on hard ticks and hard tick-borne diseases (HTBDs). In the hills and plains of western and central Nepal, the abundant cattle ticks are *Rhipicephalus (Boophilus) microplus, Haemophysalis* spp., *Ixodes* spp., and *Amblyomma* spp. [159,160]. Six species of *Haemophysalis*, five species of *Rhipicephalus*, and one species each of *Amblyomma* and *Ixodes* were reported from goats of Chitwan District [161]. Interestingly, two species of hard ticks (*Amblyomma grevaisi* and *Amblyomma varanense*) were identified in snakes of Nepal, and *Amblyomma grevaisi* was detected almost 100 years ago [162].

Similar to hard tick studies, scanty research has been carried out in Nepal on characterizing the HTBDs. In a serological study in Banke and Surkhet Districts [159], a 6.4% infection rate of TBDs was reported in cattle with *Anaplasma marginale* (5.8%) followed by *Babesia bovis* (0.6%). In Rupandehi District, the overall seroprevalence of *Coxiella burnetii* in cattle was 1.63% [163]. The percentages of *T. annulata* infection in salivary glands of *Hyalomma marginatum issaci* ticks collected from cattle raised in three Terai districts—Sunsari, Morang, and Jhapa—were 9, 27, and 21, respectively [164]. This shows a high risk of TBDs in Nepal. In a molecular study, 1.0% of *Boophilus* collected from cattle of Chitwan and Nawalparasi Districts were found positive for *Babesia* sp. infection [165].

*Rickettsia honei* was reported in one human patient infested with ticks [166]. Lyme disease (caused by the tick-transmitted spirochaete *Borrelia burgdorferi*) was reported for the first time in 2018 in Kaski District [167] and a subsequent case was reported in a patient from Gulmi District [168]. Canines transmit vector-borne diseases at the wild–domestic interface. Particularly, infections in stray dogs are alarming in Kathmandu Valley, where 81.43% of the stray dogs are infected by at least one vector-borne pathogen (*Anaplasma platys* (31%), *Babeisa vogeli* (13%), *Babesia gibsoni* (23%)) and 41.43% are co-infected with more than one vector-borne disease [169]. However, no studies have been carried out at the domestic–wildlife interface in Nepal to understand the pathways of disease transmission. Because of national and international animal trades and the intricate relationship between humans and wild animals, the spread of ticks and HTBDs is rapid. Therefore, prompt and effective preventive and control measures are needed [170].

In a recent survey conducted in September 2023, in the buffer zone communities of Shivapuri Nagarjun National Park, 65% (52 out of 80) were bitten by ticks. Among them, 56% of the respondents had the chief complaints of itching and irritation at the site of the bite, 25% of them developed rashes around the bite, 52% experienced swelling at the site of the bite, while 12.5% had fever. Furthermore, 11.25% had hemorrhage at the site of the bite which was inflicted by the tick removal and scratching against the irritation at the site of the bite while 6.25% of the tick bites were accompanied by fever. Additionally, 11.2% farmers have the perception that ticks can transmit diseases to humans while 48% of them are unaware of TBD in humans.

### 2.11. Indonesia

Geographically, Indonesia is an archipelagic country on the equator, known as having almost the most biodiversity in the world, second only to the Brazilian Amazon. The islands in eastern Indonesia are part of the Australasian continent and have different germplasm biodiversity than the western islands. The area inside of the Wallacea Webber lines holds various endemic species. In the past, research by Hoogstral, Anastos, and their colleagues contributed significantly to our understanding of tick biodiversity in Indonesia, with more than 55 tick species reported to infest different animals in the region. Some of them have particular endemic hosts, such as *Amblyomma robinsoni* of *Varanus komodoensis*, *Amblyomma babirussae* of *Babirussa babyrussa*, *Aponomma komodoense* of *Varanus komodoensis*, and *Amblyomma soembawensi* of *Varanus salvator* [171]. The most frequently reported ticks affecting livestock and companion animals include *R. sanguineus* s.l., several clades of *R. microplus* (that may result in several species after adequate studies), *Rhipicephalus pilans*, and *Haemaphysalis bispinosa* [172,173,174]. Tick-borne diseases are reported mainly from highly populated areas like Java and Bali. Tick-borne pathogens in companion animals are *Ehrlichia* sp., *Babesia* sp., and *Anaplasma* sp. whereas livestock are infected by *Babesia bigemina*, *B. bovis*, *Babesia naoakii*, *Theileria orientalis*, *Theileria* sp., *A. marginale*, and *Coxiella burnetti* [174,175,176,177,178,179]. Recently, it has become apparent that there is an alarming increase in the trading of exotic pets, primarily reptiles and amphibians, originating from Kalimantan, Sumatra, Sulawesi, Papua, and other islands. This highlights the potential for the spread of ticks and tick-borne diseases through the transportation of exotic animals. Previously, *Amblyomma* sp. was discovered in *V. salvator,* which arrived in Poland from Indonesia due to the trading of exotic animals [180]. The tick-borne bacteria *Anaplasma* spp., *Rickettsia* spp., and *Borrelia* spp. were detected in *A. varanense* infesting the lizard *V. salvator* [181]. Additionally, in Indonesia manual skin collection occurs in exotic animals such as wild snakes [182], lizards, and the Asiatic softshell turtle, *Amyda cartilaginea*. These practices highlight the importance of exercising caution and adopting appropriate measures to ensure the safety and well-being of all persons exposed to exotic species in new environments.

Tick-borne diseases pose a significant growing threat, mainly due to human activity and current climate trends. Recently, there has also been an upsurge in the number of tourists visiting remote islands in the region, leading to increased interest in these areas beyond Bali and Lombok. While the recent development progress in these regions benefits the nation’s growth, it poses a significant risk of exposure to ticks and tick-borne diseases from unknown areas that can be transmitted to new hosts. Although cases of ticks and tick-borne diseases are persistently reported, rural and urban societies pay less attention to them than mosquito-borne illnesses. An arising concern regarding tick-borne diseases occurred due to the recent lumpy skin disease outbreak from 2022–2023, with ticks considered as the vector of this massive outbreak with significant losses. Furthermore, based on our study in Central Java, more than 79% of the farmers had no awareness of zoonotic aspects of ectoparasites or arthropod-borne diseases that may harm human health, including participants with a high level of education. Zoonotic TBDs were detected in animals with pathogens such as *A. platys*, *A. marginale*, *C. burnetti*, *Borrelia* spp., and *Rickettsia* spp. [175,178,179,181,183,184,185,186]. Studies on rickettsiosis showed evidence of disease among humans, transmitted by different tick and flea genera [187,188].

Research in Indonesia shows that people tend to focus on feeding stages when dealing with ticks, neglecting off-host stages in the environment and multiple host systems. In contrast, people are widely aware of mosquitoes and their life cycles. This results in administering anti-parasitic drugs only to parasitized animals without considering appropriate treatments for the environmental stages. When anti-parasitic drug concentrations are inadequate, reinfestations can occur, and the repeated application of certain drugs can accelerate resistance to specific anti-parasitic drugs. In order to promote a better understanding of ticks and tick-borne diseases, comprehensive education and knowledge transfer should be performed on the presence of ticks, their life cycles, and the pathogens they transmit within communities.

### 2.12. Turkey

Turkey is situated at the intersection of Asia, Europe, and Africa. This unique position allows the inclusion of different climatic regions, habitat types, and animal diversity, all of which provide suitable conditions to harbor different tick species. Moreover, Turkey contains migration routes and breeding and wintering areas of many migratory birds which bring together the risk of introduction and establishment of different tick populations and associated pathogens [189,190]. More than 40 tick species have been reported in the country to date [191]. The most-recorded species were *H. marginatum, Hyalomma excavatum, Hyalomma anatolicum, Hyalomma asiaticum, Hyalomma aegyptium, R. sanguineus* s.l., *Rhipicephalus turanicus, Rhipicephalus bursa*, *Haemaphysalis parva,* and *Dermacentor marginatus* [192,193,194,195,196,197,198]. As a result of this tick species richness, a number of pathogens have been detected, including *Ehrlichia canis*, *Theileria* spp. (*Theileria ovis, T. annulata*), *Anaplasma* spp. (*A. marginale, A. phagocitophylum, A. platys, A. ovis, A. centrale, A. bovis*), *Borrelia* spp. (*B. burgdorferi* s.l., *B. turcica*), *Babesia* spp. (*Babesia ovis*, *B. bovis*, *B. bigemina*, *Babebsia major*, *Babebsia crassa, Babebsia canis*, and *B. divergens*), *Rickettsia* spp. (*Rickettsia aeschlimannii, Rickettsia hoogstraali, Rickettsia barbariae*), and *Hepatozoon canis* [199,200,201,202,203,204,205]. Furthermore, a considerable number of tick-borne viruses have been reported [206,207,208,209]. Lyme borreliosis and tick-borne encephalitis (TBE) are not prevalent in Turkey, although *I. ricinus,* the vector of these diseases, is widely distributed in the northern parts of the country [210,211,212,213].

CCHF constitutes a significant public health threat in Turkey since the first case was reported in 2002. Based on official records, 10,562 cases have been recorded from 2002–2017 and 501 of them resulted in death (https://hsgm.saglik.gov.tr/tr/zoonotikvektorel-kkka, accessed on 6 October 2023). Crimean-Congo hemorrhagic fever cases were mostly documented in rural areas in the central and northern regions of the country where agricultural and animal husbandry activities are common. Following the diagnosis of CCHF in Turkey, studies predominantly concentrated on the detection of CCHFV in ticks collected from these endemic regions [191,214,215,216,217,218]. Furthermore, CCHFV has also been recorded in eastern [219], southeastern [220], western [221], and northwestern Anatolia [204,211,222,223], which highlights the potential of emerging new endemic areas in the country.

Given that CCHF is endemic in Turkey and the number of reported cases increases annually, it is crucial to strengthen and maintain control measures such as vector control, public awareness campaigns, and vaccine development. Campaigns to inform the public about how to protect themselves against ticks should be accelerated. Additionally, it is essential to be aware of the risk factors and symptoms of CCHF to identify and diagnose probable cases early. Vaccines should be developed promptly to protect individuals at risk of exposure, including healthcare workers, veterinarians, farmers, and those residing in or traveling to endemic areas. Predicting the distribution of *H. marginatum* (the presumed main vector) is of great importance for identifying future health risks. Recent studies suggested that *H. marginatum* will remain in areas where it is currently distributed and will also expand to new areas where it has not been reported before [224]. Likewise, the progress of tick-borne encephalitis and Lyme borreliosis should be carefully monitored, as these diseases are not currently reported in Turkey but constitute a potential threat due to the presence of their tick vector. Hence, awareness-raising initiatives should not be limited only to CCHF endemic regions but expanded to the entire country.

### 2.13. Australia

Girt by sea, Australia has been in complete isolation for 40 million years. This separation has led to the evolution of 70 characterized species of argasid and ixodid ticks that have co-evolved with Australia’s unique mammalian (e.g., *Ixodes ornithorynchi*, platypus tick, and *Amblyomma triguttatum*, the ornate kangaroo tick), avian (e.g., *Argas robertsi*, Roberts’ bird tick), and reptilian (e.g., *Amblyomma albolimbatum*, stumpy-tailed lizard tick) fauna [225]. The most common biting ticks in Australia include *I. holocyclus* and *A. triguttatum* on the east and west coasts that parasitize people, respectively; *Haemaphysalis longicornis* and Rhipicephalus australis for cattle; and *R. linnaei* for dogs. It has been hypothesized that hard ticks evolved in the part of Gondwana that later became Australasia (~120 million years ago), evidenced by the basal lineage of Metastriata, Bothriocrotoninae, and Australian lineages of *Ixodes*, unique to Australia [226]. Despite Australia’s isolation, five tick species have been introduced into the Australian continent due to the movement of domestic animals following the arrival of Europeans in 1788 [227]. This introduction has led to the incursion of several tick-borne pathogens that affect companion and livestock animals in Australia, including *A. platys*, *B. vogeli*, *Borrelia persica*, *T. orientalis* complex, and more recently, *H. canis* and *E. canis*. Regarding human TBDs, only three are formally accepted: Queensland spotted fever, Flinders Island spotted fever, and Q fever [228].

The advancement of molecular techniques in recent years has led to the exponential discovery of several taxa of interest (TOIs; taxa closely related to known global tick-borne pathogens) identified within Australian ticks, wildlife, and domestic animals [229]. TOIs include *Borrelia tachyglossi* harbored within the echidna tick *Bothriocroton concolor* and several closely related unnamed *Borrelia* spp. in *Bothriocroton undatum* and within introduced and native rodents; a whole suite of *Anaplasmatacae*, *Francisellaceae*, *Midichloriaceae*, *Coxiellaeceae*, *Bartonellaceae*, *Mycoplasmatcceae*, and *Rickettsiaceae* species, including *Neoehrlichia australis* and *Neoehrlichia arcana*, *Midichloria mitochondrii, Coxiella massiliensis,* hemotropic mycoplasmas and novel species of *Anaplasma*, *Ehrlichia*, *Rickettsia*, *Francisella*; rhabdoviruses, chuviruses, coltivurses, flavivurses, and jingmenviruses; lastly, hemoprotozoa have also been recently discovered, including *Thelieria* spp., *Babesia* spp., *Trypanosoma* spp., and *Hepatozoon* spp. [229,230,231,232,233,234,235,236,237,238,239,240]. The genetic diversity of these TOIs mirrors the co-evolution of ticks and native wildlife. Furthermore, the uniqueness of these microbes answers why standard genus-specific and even species-specific assays from the northern hemisphere have failed in previous years to characterize tick-borne microbes in Australian ticks. Despite these recent discoveries, the clinical impacts on wildlife and domestic animals remain unstudied, along with whether these TOIs could be zoonotic. In addition to infectious agents, a bite from an Australian tick can lead to cutaneous reactions, envenomation of toxins resulting in paralysis in companion animals, and mammalian meat allergy in people [241].

For the past few decades, Australian patients, medical practitioners, research scientists, and the government have been occupied by the question “Does Lyme disease exist in Australia?” and subsequent controversy [241,242,243]. Despite the lack of scientific evidence for the presence of the causative agent, *Borrelia burgdorferi* s.l, and vector ticks of the *I. ricinus* group, thousands of Australians have reported suffering from non-specific arthritic, cardiological, neurological, and dermatological symptoms following a tick bite [243]. In response to the widespread public and political concern, the Australian government set up a parliamentary inquiry which led to the term debilitating symptom complexes attributed to ticks (DSCATTs), being coined to capture a range of presumptive Australian tick-borne illnesses and to differentiate them from the overseas Lyme borreliosis infection, along with the more recent guidelines for medical practitioners on treating overseas-acquired tick-borne infections [244]. Ticks are also the ultimate challenge for the Australian livestock industry, impacting all aspects of production, such as beef, dairy, and hide. The total cost of ticks and theileriosis for cattle across Australia is estimated to be AUD 161M and AUD 20M per annum, respectively [245].

Public perception of tick hosts in the media has caused much criticism around the association between bandicoots and the Australian paralysis tick (*Ixodes holocyclus*), particularly along the eastern seaboard. These unfounded claims have led to reduced efforts in bandicoot conservation, such as fox baiting, in order to control ticks [246]. Recent studies into the perception of tick encounters and wildlife observations showed that bandicoot sighting was associated with more frequent tick encounters [247]. A parallel camera trap study [248] revealed that many respondents failed to report black rat sightings, even when they were frequent. Additionally, an investigation into tick abundance [248] indicated that black rats were as responsible for tick burdens at a site level as bandicoots. This challenges preconceived biases, suggesting that blaming native bandicoot hosts over black rats for increased tick abundance in urban areas is unfair.

Some of the anticipated future challenges for the field of ticks and tick-borne diseases include predicting how climate change and anthropogenic land use will impact the density and distribution of ticks and tick-associated pathogens. These challenges will be overcome through multidisciplinary collaboration with ecologists, climate change researchers, city planners, government officials, parasitologists, and social scientists and, equally important, improved communication between medical and veterinary practitioners [249]. There is already evidence of biological changes to Australian ticks, whereby *I. holocyclus* has been identified as epizootic (temporarily prevalent) in the greater Melbourne area [250] with model predictions estimating that it could become climatically suitable for enzootic presence as early as 2030 [251]. On-going surveillance is also important for biosecurity efforts, as evident in the recent rapid spread of *Ehrlichia canis* [252]. Lastly, longitudinal studies will further elucidate the etiology and case definitions of human tick-borne diseases in Australia. For instance, studies that involve a systems biology approach using multiomic datasets should improve knowledge of the vertebrate immune responses to determine why some people and animals make full recoveries while others develop long-term debilitating sequelae [241,253]. This approach will lead to a more informed intervention for TBD, through the development of accurate biomarkers to identify susceptible patients and offer appropriate diagnosis and treatment.

## 3. Discussion, Conclusions, and Future Directions

**Figure 2:**
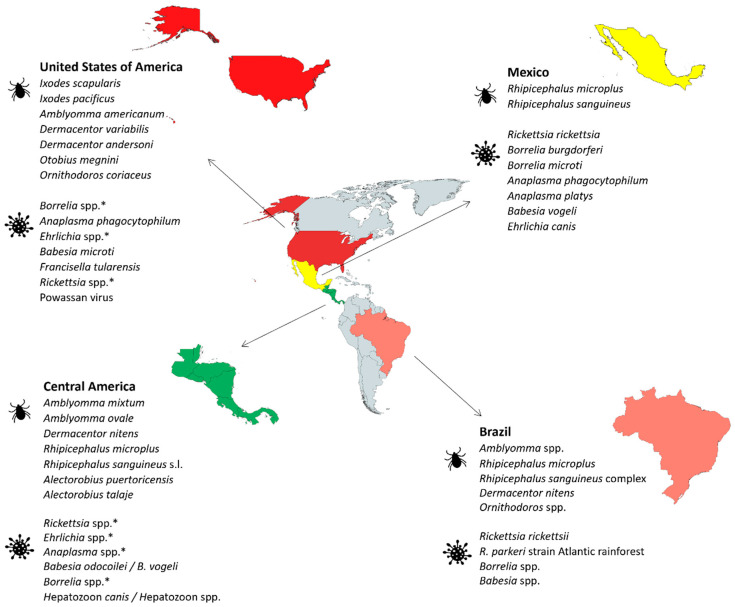
Most prevalent tick species and tick-borne pathogens in North America (USA and Mexico), Central America, and South America (Brazil). * Countries affected with more than 3 species of the same genera. Maps created with mapchart.net (https://www.mapchart.net/world.html, accessed on 6 October 2023).

**Figure 3:**
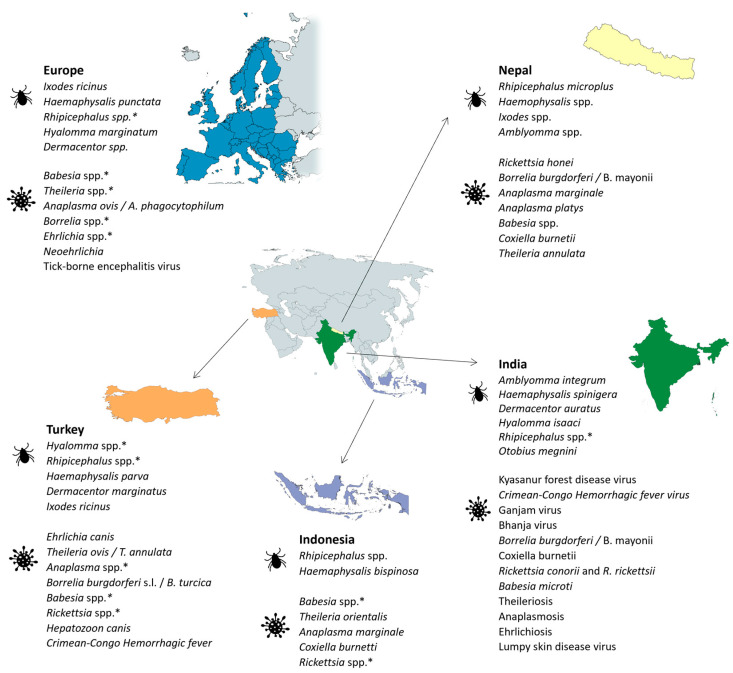
Most prevalent tick species and tick-borne pathogens in Eurasia (Europe, Turkey, India, Nepal, Indonesia). * Countries affected with more than 3 species of the same genera. Maps created with mapchart.net (https://www.mapchart.net/world.html, accessed on 6 October 2023).

**Figure 4:**
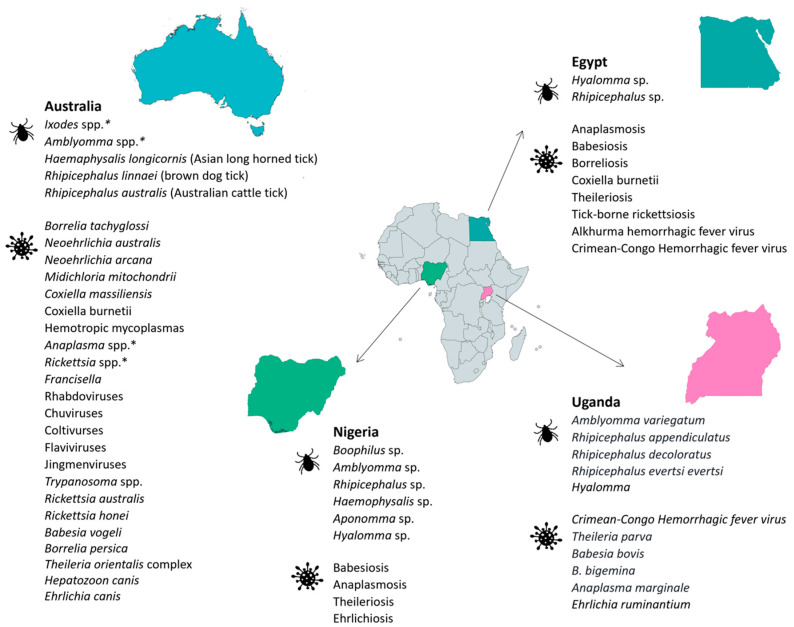
Most prevalent tick species and tick-borne pathogens in Australia and Africa (Egypt, Nigeria, and Uganda). * Countries affected with more than 3 species of the same genera. Maps created with mapchart.net (https://www.mapchart.net/world.html, accessed on 6 October 2023).

The results collected from different countries and regions worldwide are disclosed in Figure 2, Figure 3 and Figure 4 and Dataset S4. These results include published and unpublished information collected by co-authors from different countries/regions. As expected, differences in the prevalence of tick species and tick-borne pathogens are associated with geographic and climatic variables, among others. However, the information available on ticks and TBD varies between countries and regions as disclosed in Table 1 using a raw bibliometric analysis. Most of the studies (44.1%) came from Europe and the USA while Asia, North America, and Africa contributed to 36.2% of the publications. The rest of the countries/regions each contributed less than 5.5% of the publications. Reasons behind these differences may be the economic problems regarding publication in pay-to-publish journals or an obvious lack of awareness of ticks on either livestock or humans. As a rule, resource-poor countries produced fewer papers on the topic, and the contributions in this review reflect this lack of awareness.

Additionally, it should be considered that many TBDs are commonly confused with other illnesses. Therefore, patients should be aware and inform their physicians about tick exposure or the presence of ticks on domestic animals in their surroundings.

In humans, an obvious reduction of the impact of ticks and TBD could be managed by informing the population on the risks associated with ticks and TBD, involving frequent public news media and advertisements, as currently carried out in northern countries of Europe, which are measuring their impact and adaptation [254]. Although the U.S. Department of Agriculture (USDA), the National Institutes of Health (NIH), and the European Centre for Disease Prevention and Control provide online free access to information about TBD, gaps are obvious in both the transmitted information and the ability of citizens to understand the information. The same applies to ticks feeding on pets, that have an extraordinarily high contact with humans.

In accordance with bibliometric data (Table 1), differences exist between different countries and regions on the information available regarding ticks and TBD, communication to the population of the associated risks, and the implementation of vector/pathogen/disease surveillance and effective control interventions. These differences are affected by investment in science and technology.

As reported in different countries and regions, the incidence of emerging TBD will likely increase in the near future and will be recognized as studies progress in countries with fewer studies. 

Future directions should include (e.g., [112,117,241,253,255,256,257,258,259,260]) (a) systematic and comprehensive surveillance studies for ticks and TBD in both humans and animals, (b) development of innovative interventions for the control of tick infestations in domestic and wild hosts, (c) effective vaccines for controlling tick infestations in animal hosts and TBD in humans and animals, (d) implementation of regional and worldwide coordinated initiatives for more effective surveillance of ticks and TBD, detection of emerging species and diseases, and prevention of expansion worldwide, (e) application of a multidisciplinary One Health approach linking human, animal, and environmental health, (f) monitoring acaricide application and resistance in different regions, (g) transgenic and paratransgenic interventions in both hosts and ticks to control ticks and TBD, (h) modeling the effect of climate change and anthropogenic land use on the possible expansion of wild hosts and tick populations and incidence of TBD, (i) application of multiomic system biology approaches to the study of host immune-mediated mechanisms and identification of biomarkers in susceptible patients and animal hosts for efficacious disease diagnosis and treatment, and (j) communication to the general population and healthcare system of the risks associated with ticks and TBD and measures to reduce these risks.

## Figures and Tables

**Table 1:** Bibliometric analysis on ticks and TBD.

Terms of Search	Number of Publications (%)
Tick AND Tick-borne disease	21,302; corrected as 21,715
Tick AND Tick-borne disease AND Europe	5194 (23.9%)
Tick AND Tick-borne disease AND USA	4394 (20.2%)
Tick AND Tick-borne disease AND Asia	2950 (13.6%)
Tick AND Tick-borne disease AND North America	2935 (13.5%)
Tick AND Tick-borne disease AND Africa	1967 (9.1%)
Tick AND Tick-borne disease AND China	1119 (5.2%)
Tick AND Tick-borne disease AND Russia	902 (4.2%)
Tick AND Tick-borne disease AND South America	717 (3.3%)
Tick AND Tick-borne disease AND Brazil	703 (3.2%)
Tick AND Tick-borne disease AND Australia	377 (1.7%)
Tick AND Tick-borne disease AND Mexico	267 (1.2%)
Tick AND Tick-borne disease AND Central America	190 (0.9%)

Search was conducted on PubMed (https://pubmed.ncbi.nlm.nih.gov, accessed on 14 September 2023). Results for “Tick” AND “Tick-borne disease” were corrected as the total number of entries for the rest of the rows. Percentages were calculated based on the corrected total number of entries.

## Data Availability

All data are disclosed in the paper and Supplementary Materials.

## Supplementary Materials

The following supporting information can be downloaded at: https://www.mdpi.com/article/10.3390/pathogens12101258/s1, Dataset S1: USDA and NIH online freely accessible information about TBD; Dataset S2: CDC online information on TBD surveillance; Dataset S3: Tick-borne pathogens identified in ticks from Central America; Dataset S4: Distribution of ticks and tick-borne pathogens worldwide.
