# Supplementary material for: Perception of Ticks and Tick-Borne Diseases Worldwide
Source: Pathogens. 2023 Oct 19;12(10):1258. doi: 10.3390/pathogens12101258 (PMC10610181; doi:10.3390/pathogens12101258)
Supplement: Supplementary file 1 [file pathogens-12-01258-s001.zip › pathogens-2672993-supplementary.pdf]

Supplementary Materials: Dataset S1. USDA and NIH online free access information about TBD. **Dataset S2.** CDC online information on TBD surveillance. **Dataset S3.** Tick-borne pathogens identified in ticks from Central America.

Dataset S1. USDA and NIH online free access information about TBD.

U.S. Department of Agriculture (USDA). Animal and Plant Health Inspection Service. Vector-borne diseases. Last Modified: Jul 26, 2022. https://www.aphis.usda.gov/aphis/ourfocus/animalhealth/animal-disease-information/cattle-disease-information/cattle-vector-borne-diseases

U.S. Department of Agriculture (USDA). National Institute of Food and Agriculture. May 5, 2022. Research tackling Lyme disease and other tick-borne diseases. https://www.nifa.usda.gov/about-nifa/blogs/research-tackling-lyme-disease-other-tick-borne-diseases

National Institutes of Health (NIH). National Institute of Allergy and Infectious Diseases. October 10, 2019. Tick-borne diseases. https://www.niaid.nih.gov/diseases-conditions/tick-borne-diseases

National Institutes of Health (NIH). National Institute of Allergy and Infectious Diseases. October 18, 2017. Tick-borne Disease-Specific Research. https://www.niaid.nih.gov/diseases-conditions/tick-borne-diseases-specific

NIH tick-borne diseases strategic planning team. National Institutes of Health (NIH). October 9, 2019. NIH strategic plan for tick-borne disease research. https://www.niaid.nih.gov/sites/default/files/NIH-Strategic-Plan-Tick-borne-Disease-Research-2019.pdf

National Institutes of Health (NIH). News in Health. June 2012. An Uptick in Ticks. Don't Let Ticks Make You Sick. https://newsinhealth.nih.gov/2023/06/uptick-ticks

National Institutes of Health (NIH). News in Health. May 2014. Tick Talk. Block tick bites and Lyme disease. https://newsinhealth.nih.gov/2014/05/tick-talk

Dataset S2. CDC online information on TBD surveillance.
https://www.cdc.gov/ticks/data-summary/dashboard-data-files/Selected_Tick-borne_Diseases_United-States.csv
Year,Count,Disease
2016,36429,Lyme disease
2016,4151,Anaplasmosis
2016,4269,Spotted Fever Rickettsiosis
2016,1910,Babesiosis
2016,1377,Ehrlichia chaffeensis ehrlichiosis
2016,230,Tularemia
2016,200,Undetermined ehrlichiosis/anaplasmosis
2016,22,Ehrlichia ewingii ehrlichiosis
2016,22,Powassan virus disease
2017,42743,Lyme disease
2017,5762,Anaplasmosis
2017,6248,Spotted Fever Rickettsiosis
2017,2368,Babesiosis
2017,1642,Ehrlichia chaffeensis ehrlichiosis
2017,239,Tularemia
2017,269,Undetermined ehrlichiosis/anaplasmosis
2017,45,Ehrlichia ewingii ehrlichiosis
2017,34,Powassan virus disease
2018,33666,Lyme disease
2018,4008,Anaplasmosis
2018,5544,Spotted Fever Rickettsiosis
2018,2160,Babesiosis
2018,1799,Ehrlichia chaffeensis ehrlichiosis
2018,229,Tularemia
2018,283,Undetermined ehrlichiosis/anaplasmosis
2018,33,Ehrlichia ewingii ehrlichiosis
2018,21,Powassan virus disease
2019,34945,Lyme disease
2019,5655,Anaplasmosis
2019,5207,Spotted Fever Rickettsiosis
2019,2420,Babesiosis
2019,2093,Ehrlichia chaffeensis ehrlichiosis
2019,274,Tularemia
2019,185,Undetermined ehrlichiosis/anaplasmosis
2019,43,Ehrlichia ewingii ehrlichiosis
2019,43,Powassan virus disease
https://www.cdc.gov/ticks/data-summary/dashboard-data-files/total_reported_cases_1619.csv
Year,2016,2017,2018,2019
Reported Tick-borne Disease,Count,Count,Count,Count
Lyme disease,"36,429","42,743","33,666","34,945"
Anaplasmosis,"4,151","5,762","4,008","5,655"
Spotted Fever Rickettsiosis,"4,269","6,248","5,544","5,207"
Babesiosis,"1,910","2,368","2,160","2,420"
Ehrlichia chaffeensis ehrlichiosis,"1,377","1,642","1,799","2,093"
Tularemia,230,239,229,274
Undetermined ehrlichiosis/anaplasmosis,200,269,283,185

Ehrlichia ewingii ehrlichiosis,22,45,33,43
Powassan virus disease,22,34,21,43
Total,"48,610","59,350","47,743","50,865"

Dataset S3. Tick-borne pathogens identified in ticks from Central America.

Country	*Rickettsia* spp.	Arthropod species
Belize	*Rickettsia amblyommatis*	*Amblyomma pacae*
		Amblyomma cajennense s.l.
		Amblyomma ci. *maculatum*
	Rickettsia parkeri	*Amblyomma maculatum*
	Rickettsia st. Atlantic Rainforest	*Amblyomma ovale*
	Rickettsia sp.	*Ixodes affinis*
		Rhipicephalus sanguineus s.l.
		Amblyomma ovale
Costa Rica	*Rickettsia amblyommatis*	*Amblyomma geayi*
		Amblyomma longirostre
		Amblyomma mixtum
		Amblyomma ovale
		Amblyomma cf. *parvum*
		Amblyomma spp.
		Dermacentor nitens
		Rhipicephalus sanguineus s.l.
		Amblyomma geayi
	Rickettsia bellii	*Amblyomma sabanerae*
	Rickettsia rickettsii	*Amblyomma mixtum*
		Amblyomma varium
		Haemaphysalis leporispalustris
	Rickettsia sp. st. IbR/CRC	*Ixodes* ci. *boliviensis*
	Rickettsia sp.	*Ixodes* cf. *minor*
		Amblyomma ovale
		Rhipicephalus microplus
	Rickettsia rhipicephali	*Dermacentor latus*
	Ca. "Rickettsia nicoyana"	*Ornithodoros knoxjonesi*
	Rickettsia sp. st. Colombianensi	*Amblyomma dissimile*
	Rickettsia sp. ci. raoultii	*Amblyomma geayi*
El Salvador	*Rickettsia amblyommatis*	*Amblyomma mixtum*
		Amblyomma cf. *parvum*
		Dermacentor nitens
		Rhipicephalus sanguineus s.l.
	Rickettsia bellii	*Amblyomma sabanerae*
		Amblyomma ovale
	Rickettsia parkeri	*Amblyomma ovale*
	Ca. "R. Colomnianensis"	*Amblyomma dissimile*
		Amblyomma scutatum
	Rickettsia sp.	*Amblyomma cajennense* s.l.
Honduras	*Rickettsia amblyommatis*	*Amblyomma longirostre*
		Amblyomma mixtum
	Rickettsia st. Colombianensi	*Amblyomma dissimile*
Nicaragua	*Rickettsia amblyommatis*	*Amblyomma sculptum*
	Rickettsia africae	*Amblyomma ovale*
	Rickettsia st. Aragoi	*Amblyomma triste*
	Rickettsia spp.	*Amblyomma maculatum*
		Dermacentor dissimilis
		Rhipicephalus sanguineus s.l.
Panamá	*Rickettsia amblyommatis*	*Amblyomma auricularium*
		Amblyomma geayi
		Amblyomma longirostre
		Amblyomma mixtum
		Amblyomma ovale
		Amblyomma pacae
		Amblyomma varium
		Dermacentor nitens
		Haemaphysalis juxtakochi

		Rhipicephalus sanguineus s.l.
		Ctenocephalides felis
	Rickettsia bellii	*Amblyomma sabanerae*
		Amblyomma dissimile
		Amblyomma rotundatum
		Amblyomma mixtum
	Rickettsia rickettsii	*Amblyomma mixtum*
		Rhipicephalus sanguineus s.l.
		Dermacentor nitens
	Ca. "R. Colomnianensis"	*Amblyomma dissimile*
	Rickettsia sp.	*Ixodes affinis*
	Rickettsia sp.	*Ixodes* cf. *boliviensis*
	Rickettsia sp.	*Ixodes tapirus*
	Rickettsia sp. ci. raoultii	*Amblyomma geayi*
	Rickettsia sp. ci. tamurae	*Amblyomma dissimile*

Country	Anaplasmataceae	Arthropod species
Costa Rica	*Ehrlichia canis*	*Rhipicephalus sanguineus* s.l.
Nicaragua		
Panamá	*Anaplasma marginale*	*Rhipicephalus microplus*
	Anaplasma platys	*Rhipicephalus sanguineus* s.l.
	Anaplasma phacocitophilum	*Ixodes tapirus*
		Rhipicephalus microplus
	Ehrlichia canis	*Rhipicephalus sanguineus* s.l.
	Ehrlichia ci. *chaffeensis*	*Amblyomma mixtum*
		Dermacentor nitens

Country	*Borrelia*	Arthropod species
Panamá	*Borrelia recurrentis* s.l.	*Ornithodoros rudis*
		Alecterobius talaje
	Borrelia puertoricensis	*Alecterobius puertoricensis*
	Borrelia burgdorferi group	*Ixodes* cf. *boliviensis*

Country	Hemoparasite	Arthropod species
Panama	*Hepatozoon* spp.	*Ixodes* cf. *boliviensis*
	Babesia odocoilei	*Ixodes* cf. *boliviensis*

Selected references in support to Dataset S3

Ábrego-Sánchez, L., Jiménez-Rocha, A.E., Montenegro, V.M., Dolz, G. Detección de agentes zoonóticos (*Ehrlichia* spp., *Rickettsia* spp., *Anaplasma platys* y *Borrelia burgdorferii* s.l.) en garrapatas recolectadas de perros en Costa Rica (Detection of zoonotic agents in ticks collected from dogs in Costa Rica). Fourth Latin American Congress on Rickettsial Diseases, San José, Costa Rica. *Acta Méd. Costarric*. 2013;55:88.

Bermúdez CSE, Troyo A. A review of the genus *Rickettsia* in Central America. *Res Rep Trop Med*. 2018;9:103-112. doi:10.2147/RRTM.S160951

Bermúdez C SE, Félix ML, Domínguez A L, Kadoch N, Muñoz-Leal S, Venzal JM. Molecular screening for tick-borne bacteria and hematozoa in *Ixodes* cf. *boliviensis* and *Ixodes tapirus* (Ixodida: Ixodidae) from western highlands of Panama. *Curr Res Parasitol Vector Borne Dis*. 2021;1:100034. doi:10.1016/j.crpvbd.2021.100034

Bermúdez S, Martínez-Mandiche J, Domínguez L, et al. Diversity of Rickettsia in ticks collected from wild animals in Panama. *Ticks Tick Borne Dis*. 2021;12(4):101723. doi:10.1016/j.ttbdis.2021.101723

Dunn LH, Clark HC. Notes on relapsing fever in Panama with special reference to animal hosts. *Am. J. Trop. Med. Hyg*. 1933;13:201-209.

Bermúdez SE, Armstrong BA, Domínguez L, et al. Isolation and genetic characterization of a relapsing fever spirochete isolated from *Ornithodoros puertoricensis* collected in central Panama. *PLoS Negl Trop Dis*. 2021;15(8):e0009642. doi:10.1371/journal.pntd.0009642

Orgzewalska M, Bermúdez S. Detección molecular de Rickettsia bellii en *Amblyomma rotundatum* Koch 1884 (Ixodida: Ixodidae) en Panamá. Tecnociencia 2019;21(1):41-56.

Romero L, Costa FB, Labruna MB. Ticks and tick-borne Rickettsia in El Salvador. *Exp Appl Acarol*. 2021;83(4):545-554. doi:10.1007/s10493-021-00610-w

Romero LE, Binder LC, Marcili A, Labruna MB. Ticks and tick-borne rickettsiae from dogs in El Salvador, with report of the human pathogen *Rickettsia parkeri*. *Ticks Tick Borne Dis*. 2023;14(5):102206. doi:10.1016/j.ttbdis.2023.102206

Springer A, Montenegro VM, Schicht S, et al. Detection of *Rickettsia monacensis* and *Rickettsia amblyommatis* in ticks collected from dogs in Costa Rica and Nicaragua. *Ticks Tick Borne Dis*. 2018;9(6):1565-1572. doi:10.1016/j.ttbdis.2018.08.002

Dataset S4. Distribution of ticks and tick-borne pathogens worldwide.

Country	Most recorded Tick specie	Pathogens
**United States of America**	Human:<br>*Ixodes scapularis*<br>*Amblyomma americanum*<br>*Dermacentor variabilis*<br>*Ixodes pacificus*<br>*Dermacentor andersoni*<br>*Otobius megnini*<br>*Ornithodoros coriaceus*<br><br>*Other:*<br>*Ixodes cookei*, *Dermacentor occidentalis*, *Rhipicephalus sanguineus* sensu lato, *Dermacentor albipictus*, and *Amblyomma maculatum*<br><br><br><br>Cattle:<br>*Rhipicephalus annulatus*<br>*Rhipicephalus microplus*<br>*Haemaphysalis longicornis* (Asian longhorned ticks)<br>*Ambylomma hebraeum* and *Amblyomma variegatum (*Bont and Tropical Bont Ticks)	*Borrelia burgdorferi* sensu stricto and *Borrelia mayonii* (Lyme disease)<br>*Borrelia miyamotoi* (hard tick-borne relapsing fever)<br>*Anaplasma phagocytophilum* (human anaplasmosis)<br>*Ehrlichia muris eauclairensis, Ehrlichia chaffeensis*, *Ehrlichia ewingii* (ehrlichiosis)<br>*Babesia microti* (babesiosis)<br>*Babesia duncani*<br>*Francisella tularensis* (Tularemia)<br>*Borrelia hermsii*, *B. turicata*, *B. parkeri*<br>*Rickettsia rickettsii* (Rocky Mountain spotted fever)<br>*Rickettsia philipii* (Pacific Coast tick fever)<br>*Rickettsia parkeri* (other spotted fever)<br>Powassan virus (Powassan encephalitis)<br>Colorado tick fever virus (Colorado tick fever)<br>Bourbon vírus<br>Heartland vírus<br><br>Cattle:<br>Bovine babiesiosis and anaplasmosis<br>*Theileria orientalis Ikeda*<br>*Ehrlichia ruminantium*<br>Nairobi sheepdisease
**Alasca**	**Native species**<br>*Haemaphysalis leporispalustris*<br>*Ixodes angustus*<br>*Ixodes auritulus*<br>*Ixodes howelli*<br>*Ixodes signatus*	

	Ixodes uriae<br><br>**Non-native species**<br>*A. americanum*<br>*Dermacentor andersoni*<br>*D. occidentalis*<br>*Dermacentor variabilis*<br>*Ixodes pacificus*<br>*Ixodes ricinus*<br>*Ixodes scapularis*<br>*Ixodes texanus*<br>*Rhipicephalus sanguineus* s.l.	
**Mexico**	***Most common***:<br>*Rhipicephalus microplus*, *Rhipicephalus sanguineus*<br><br>***Other:***<br>*Amblyomma* spp., *Dermacentor* spp., *Ixodes* spp., *Otobius megnini*	**Human:**<br>*Rickettsia rickettsia* (Rickettsiosis)<br>*B. burgdorferi*<br>*B. microti*<br>*A. phagocytophilum*<br><br>**Cattle:**<br>Babesiosis and Anaplasmosis<br><br>**Horses:**<br>Equine babesiosis and Theileriosis<br><br>**Dogs:**<br>*Canine babesiosis (B. vogeli)*<br>*Ehrlichia canis*<br>*Anaplasma platys*<br>*A. phagocytophilum*
**Central America**	*Amblyomma mixtum*<br>*Amblyomma ovale*<br>*Dermacentor nitens*	Rickettsiosis (*Rickettsia rickettsii)*<br><br>Others:

	Rhipicephalus microplus<br>*Rhipicephalus sanguineus* s.l.<br>*Alectorobius puertoricensis*<br>*Alectorobius talaje*	Ehrlichiosis (*E. canis, E.* cf. *chaffeensis)*<br>Anaplasmosis (*A. marginale, A. phagocitophylum, A. platys)*<br>Relapsing fever<br>Babesiosis (*B. odocoilei, B. vogeli)*<br>*Borrelia (B. puertoricensis*, *Borrelia burgdorferi* group)<br>Hepatozoon (*H. canis*, *H*epatozoon spp)
**Guatemala**	*Ornithodoros talaje*<br>*Amblyomma sabanerae*	
**Panamá**	*Amblyomma auricularium*<br>*Amblyomma geayi*<br>*Amblyomma longirostre*<br>*Amblyomma mixtum*<br>*Amblyomma ovale*<br>*Amblyomma pacae*<br>*Amblyomma varium*<br>*Amblyomma sabanerae*<br>*Amblyomma dissimile*<br>*Amblyomma rotundatum*<br>*Dermacentor nitens*<br>*Haemaphysalis juxtakochi*<br>*Rhipicephalus sanguineus s.l.*<br>*Ctenocephalides felis*<br>*Ixodes affinis*<br>*Ixodes cf. boliviensis*<br>*Ixodes tapirus*<br>*Ornithodoros rudis*<br>*Alecterobius talaje*<br>*Alecterobius puertoricensis*	*Rickettsia amblyommatis*<br>*Rickettsia bellii*<br>*Rickettsia rickettsii*<br>*Ca. "R. Colomnianensis"*<br>*Rickettsia sp.*<br>*Rickettsia sp. ci. raoultii*<br>*Rickettsia sp. ci. Tamurae*<br>*Anaplasma marginale*<br>*Anaplasma platys*<br>*Anaplasma phacocitophilum*<br>*Ehrlichia canis*<br>*Ehrlichia ci. chaffeensis*<br>*Borrelia recurrentis s.l.*<br>*Borrelia puertoricensis*<br>*Borrelia burgdorferi group*<br>*Hepatozoon spp.*<br>*Babesia odocoilei*
**Costa Rica**	*Amblyomma geayi*	*Rickettsia amblyommatis*

	Amblyomma longirostre<br>*Amblyomma mixtum*<br>*Amblyomma ovale*<br>*Amblyomma cf. parvum*<br>*Amblyomma spp.*<br>*Amblyomma dissimile*<br>*Amblyomma sabanerae*<br>*Amblyomma varium*<br>*Dermacentor nitens*<br>*Dermacentor latus*<br>*Rhipicephalus sanguineus s.l.*<br>*Rhipicephalus microplus*<br>*Haemaphysalis leporispalustris*<br>*Ixodes ci. boliviensis*<br>*Ixodes cf. minor*<br>*Ornithodoros knoxjonesi*	*Rickettsia bellii*<br>*Rickettsia rickettsii*<br>*Rickettsia sp. st. IbR/CRC*<br>*Rickettsia sp.*<br>*Rickettsia rhipicephali*<br>*Ca. "Rickettsia nicoyana"*<br>*Rickettsia sp. st. Colombianensi*<br>*Rickettsia sp. ci. raoultii*<br>*Ehrlichia canis*
**Belize**	*Amblyomma pacae*<br>*Amblyomma cajennense s.l.*<br>*Amblyomma ci. maculatum*<br>*Amblyomma maculatum*<br>*Amblyomma ovale*<br>*Ixodes affinis*<br>*Rhipicephalus sanguineus s.l.*	*Rickettsia amblyommatis*<br>*Rickettsia parkeri*<br>*Rickettsia st. Atlantic Rainforest*<br>*Rickettsia sp.*
**El Salvador**	*Amblyomma mixtum*<br>*Amblyomma cf. parvum*<br>*Dermacentor nitens*<br>*Rhipicephalus sanguineus s.l.*<br>*Amblyomma sabanerae*<br>*Amblyomma ovale*<br>*Amblyomma dissimile*<br>*Amblyomma scutatum*<br>*Amblyomma cajennense s.l.*	*Rickettsia amblyommatis*<br>*Rickettsia bellii*<br>*Rickettsia parkeri*<br>*Ca. "R. Colomnianensis"*<br>*Rickettsia sp.*

**Honduras**	*Amblyomma longirostre*<br>*Amblyomma mixtum*<br>*Amblyomma dissimile*	*Rickettsia amblyommatis*<br>*Rickettsia st. Colombianensi*
**Brazil**	Human:<br>*Amblyomma sculptum* larvae and nymphs<br>*Amblyomma aureolatum*<br>*Ornithodoros* spp.<br><br>Cattle:<br>*Rhipicephalus microplus*<br><br>*Horse:*<br>*Amblyomma sculptum* and *Dermacentor nitens*<br><br>Dogs:<br>*Rhipicephalus sanguineus* complex (*Rhipicephalus linnaei*)<br>*Amblyomma aureolatum*<br><br>Wild carnivores:<br>*Amblyomma aureolatum*<br>*Amblyomma ovale*<br>*Amblyomma tigrinum*	Human:<br>*Rickettsia rickettsii* (Brazilian Spotted-Fever)<br>*Rickettsia parkeri* strain Atlantic rainforest<br>*Borrelia* spp.<br><br>Cattle:<br>*Babesia* spp. and *Anaplasma marginale* (Bovine parasitic sadness)<br><br>Horses:<br>*Babesia caballi* and *Theileria equi (*Equine Piroplasmosis)<br><br>Dogs:<br>*Ehrliquia canis* (canine monocytic ehrlichiosis)<br>*Babesia vogeli (*canine piroplasmosis)<br>*Rickettsia rickettsii*<br>*Rangelia vitalii (*canine rangeliosis)<br>Hepatozoidae species (e.g., *Hepatozoon canis)*<br><br>Wild carnivores:<br>*Rickettsia rickettsii*<br>*Rickettsia parkeri* Atlantic rainforest strain<br>*Rickettsia parkeri* sensu stricto
**Europe**	*Ixodes ricinus*<br>*Haemaphysalis punctata*<br>*Rhipicephalus spp.*<br>*Hyalomma marginatum*<br>*Dermacentor spp.*	Ruminants and carnivores:<br>*Babesia* spp.<br>*Theileria* spp.<br>*Anaplasma ovis*<br>*Anaplasma phagocytophilum*

		Borrelia spp.<br>*Ehrlichia* spp.<br>*Neoehrlichia*<br>Tick-borne encephalitis virus
**Egypt**	*Hyalomma* sp.<br>*Rhipicephalus* sp.	Humans:<br>Anaplasmosis<br>Borreliosis<br>Coxiella burnetii (Q fever)<br>Tick-borne rickettsiosis<br>Babesiosis<br>Alkhurma hemorrhagic fever<br>Crimean-Congo Hemorrhagic fever<br><br>Livestock:<br>Anaplasmosis<br>Babesiosis<br>Theileriosis<br>Q fever
**Uganda**	Human:<br>Hyalomma<br><br>Cattle:<br>*Rhipicephalus appendiculatus*<br>*Rhipicephalus decoloratus*<br>*Amblyomma variegatum*<br>*Rhipicephalus evertsi evertsi*	Human:<br>*Crimean-Congo Hemorrhagic fever* virus<br><br>Cattle:<br>*Theileria parva*<br>*Babesia bovis*<br>*B. bigemina*<br>*Anaplasma marginale*<br>*Ehrlichia ruminantium*
**Nigeria**	*Boophilus* sp.<br>*Amblyomma* sp.<br>*Rhipicephalus* sp.<br>*Haemophysalis* sp.<br>*Aponomma* sp.	Babesiosis<br>Anaplasmosis<br>Theileriosis<br>Ehrlichiosis

	Hyalomma sp.	
**India**	Humans:<br>*Amblyomma integrum*<br>*Haemaphysalis spinigera*<br>*Dermacentor auratus*<br>*Hyalomma isaaci*<br>*Rhipicephalus haemaphysaloides*<br>*Rhipicephalus sanguineus* s.l.<br>*Otobius megnini*<br><br>Cattle:<br>*Rhipicephalus microplus*	Human:<br>Kyasanur forest disease virus<br>*Crimean-Congo Hemorrhagic fever* virus<br>Ganjam virus<br>Bhanja virus<br>*Borrelia burgdorferi* and B. mayonii (Lyme disease)<br>Coxiella burnetii (Q fever)<br>*Rickettsia conorii* and *R. rickettsii*)<br>*Babesia microti* (Babesiosis)<br><br>Animals:<br>Theileriosis<br>Babesiosis<br>Anaplasmosis<br>Ehrlichiosis<br>Lumpy skin disease virus
**Nepal**	Cattle:<br>*Rhipicephalus microplus*<br>*Haemophysalis* spp.<br>*Ixodes* spp.<br>*Amblyomma* spp.<br><br>Goats:<br>*Haemophysalis* spp.<br>*Rhipicephalus* spp.<br>*Amblyomma* sp.<br>*Ixodes* sp.	Human:<br>*Rickettsia honei*<br>*Borrelia burgdorferi* and B. mayonii (Lyme disease)<br><br>Cattle:<br>*Anaplasma marginale*<br>*Babesia bovis*<br>*Coxiella burnetii*<br>*Theileria annulata*<br><br>Dogs:<br>*Anaplasma platys*<br>*Babeisa vogeli*<br>*Babesia gibsoni*
**Indonesia**	*Varanus komodoensis:*	*Varanus komodoensis:*

	Amblyomma robinsoni<br>*Aponomma komodoense*<br><br>*Babirussa babyrussa:*<br>*Amblyomma babirussae*<br><br>*Varanus salvator:*<br>*Amblyomma soembawensi*<br><br>Livestock and Companion animals:<br>*Rhipicephalus sanguineus* s.l.<br>*Rhipicephalus microplus*<br>*Rhipicephalus pilans*<br>*Haemaphysalis bispinosa*	*Anaplasma* spp., *Rickettsia* spp., and *Borrelia* spp.<br><br>Livestock:<br>*Babesia bigemina*<br>*Babesia bovis*<br>*Babesia naoakii*<br>*Theileria orientalis*<br>*Theileria* sp.<br>*A. marginale*<br>*Coxiella burnetti*<br><br>Companion animals:<br>*Ehrlichia* sp.<br>*Babesia* sp.<br>*Anaplasma* sp.
**Turkey**	*Hyalomma marginatum*<br>*Hyalomma excavatum*<br>*Hyalomma anatolicum*<br>*Hyalomma asiaticum*<br>*Hyalomma aegyptium*<br>*Rhipicephalus sanguineus* s.l.<br>*Rhipicephalus turanicus*<br>*Rhipicephalus bursa*<br>*Haemaphysalis parva*<br>*Dermacentor marginatus*<br>*Ixodes ricinus*	*Ehrlichia canis*<br>*Theileria* spp. (*Theileria ovis, T. annulata*)<br>*Anaplasma* spp. (*A. marginale, A. phagocitophylum, A. platys, A. ovis, A. centrale, A. bovis*)<br>*Borrelia* spp. (*B. burgdorferi* s.l., *B. turcica*)<br>*Babesia* spp. (*Babesia ovis*, *B. bovis*, *B. bigemina*, *Babebsia major*, *Babebsia crassa, Babebsia canis* and *B. divergens*)<br>*Rickettsia* spp. (*Rickettsia aeschlimannii, Rickettsia hoogstraali, Rickettsia barbariae*)<br>*Hepatozoon canis*<br>*Crimean-Congo Hemorrhagic fever* virus
**Australia**	Mammalian:<br>*Ixodes ornithorynchi (*platypus tick)<br>*Amblyomma triguttatum* (ornate kangaroo tick)<br><br>Avian:	***New pathogens:***<br>*Borrelia tachyglossi*<br>*Neoehrlichia australis*<br>*Neoehrlichia arcana*<br>*Midichloria mitochondrii*

Argas robertsi	*Coxiella massiliensis*
	hemotropic mycoplasmas
Reptilian:	*Anaplasma* spp.
Amblyomma albolimbatum	*Ehrlichia* spp.
	Rickettsia spp.
Ixodes holocyclus	*Francisella*
Amblyomma triguttatum	Rhabdoviruses
Haemaphysalis longicornis	Chuviruses
	Coltivurses
	Flavivirus
	Jingmenviruses
	Thelieria spp.
	Babesia spp.
	Trypanosoma spp.
	Hepatozoon spp
	Humans:
	Rickettsia australis (Queensland spotted fever)
	Rickettsia honei (Flinders Island spotted fever)
	Coxiella burnetii (Q Fever)
	Livestock and Companion animals*:*
	Anaplasma platys
	A. marginale
	Babesia bovis
	B. bigemina
	B. vogeli
	Borrelia persica
	T. orientalis complex
	H. canis
	E. canis
